# Supplementary material for: Fluorocarbon Plasma-Polymerized Layer Increases the Release Time of Silver Ions and the Antibacterial Activity of Silver-Based Coatings
Source: Nanomaterials (Basel). 2024 Mar 29;14(7):609. doi: 10.3390/nano14070609 (PMC11013325; doi:10.3390/nano14070609)
Supplement: Supplementary file 1 [file nanomaterials-14-00609-s001.zip › nanomaterials-2833027-supplementary.pdf]

Fluorocarbon plasma-polymerized layer increases the release time of silver ions and the antibacterial activity of silver-based coatings

Linda Bonilla-Gameros [1], Pascale Chevallier [1], Xavier Delvaux[2], Lidi Astrid Yáñez-Hernández[1], Laurent Houssiau[2], Xavier Minne[3], Vanessa P. Houde[3], Andranik Sarkissian[4] and Diego Mantovani[1]

[1] Laboratory for Biomaterials and Bioengineering, (CRC-Tier I), Dept of Min-Met-Materials Eng and Regenerative Medicine, CHU de Quebec, Laval University, Quebec City, Canada

[2] Laboratoire Interdisciplinaire de Spectroscopie Electronique, Namur Institute of Structured Matter, University of Namur, 61 Rue de Bruxelles, 5000 Namur, Belgium

[3] Oral Ecology Research Group (GREB), Faculty of Dentistry, Université Laval, 2420 rue de la Terrasse, Québec, QC G1V 0A6, Canada

[4] Plasmionique Inc, 171-1650 Boul Lionel Boulet, Varennes, QC J3X1S2, Canada

Supplementary data

Figure S1: a) High resolution XPS spectra of Ag 3d and b) AES spectra of Ag MNN region for a-C:H:Ag (top) and a-C:H:AgO (bottom).

Figure S2: WCA images of the uncoated and CF_x coated a-C:H samples depending on the duty cycle used and their respective WCA values.

Figure S3: Particle size distribution and AFM images of a) a-C:H:Ag and b) a-C:H:AgO coatings before CF_x deposition.

Figure S4: Percentage of *E. coli* growth after 4 h in contact with Si (as negative control), 5.3CF_x/a-C:H:Ag, and 5.3CF_x/a-C:H:AgO.

High resolution of X-ray photoelectron spectroscopy (XPS) of Ag 3d spectra and Auger electron spectroscopy (AES) of Ag MNN region were used to analyze the chemical states of Ag in a-C:H:Ag and a-C:H:AgO samples before the deposition of the CF_x film. The fittings and positions of Ag 3d components ($3d^{3/2}$ and $3d^{5/2}$) are shown in **Figure S1a.** The main peak attributed to Ag-Ag bond was fitted at 368.40 ± 0.06 eV for a-C:H:Ag and at 368.42 ± 0.07 eV for a-C:H:AgO with obtained chi-square (x^2) values of 1.6 ± 0.2 and 1.3 ± 0.1, respectively. In this sense, the Ag MNN Auger spectra (**Figure S1b**) for a-C:H:Ag and a-C:H:AgO were studied and showed the two principal Auger transitions of each structure assigned to Ag $M_5N_{45}N_{45}$ and Ag $M_4N_{45}N_{45}$. In this sense, the main differences between both samples lie in the full width at half maximum (FWHM) of the Ag $3d^{5/2}$ peak and the value of the modified Auger parameter (α′): 1.07 ± 0.01 eV and 725.5 ± 0.2 eV for a-C:H:Ag, 1.15 ± 0.02 eV and 724.8 ± 0.5 eV for a-C:H:AgO.

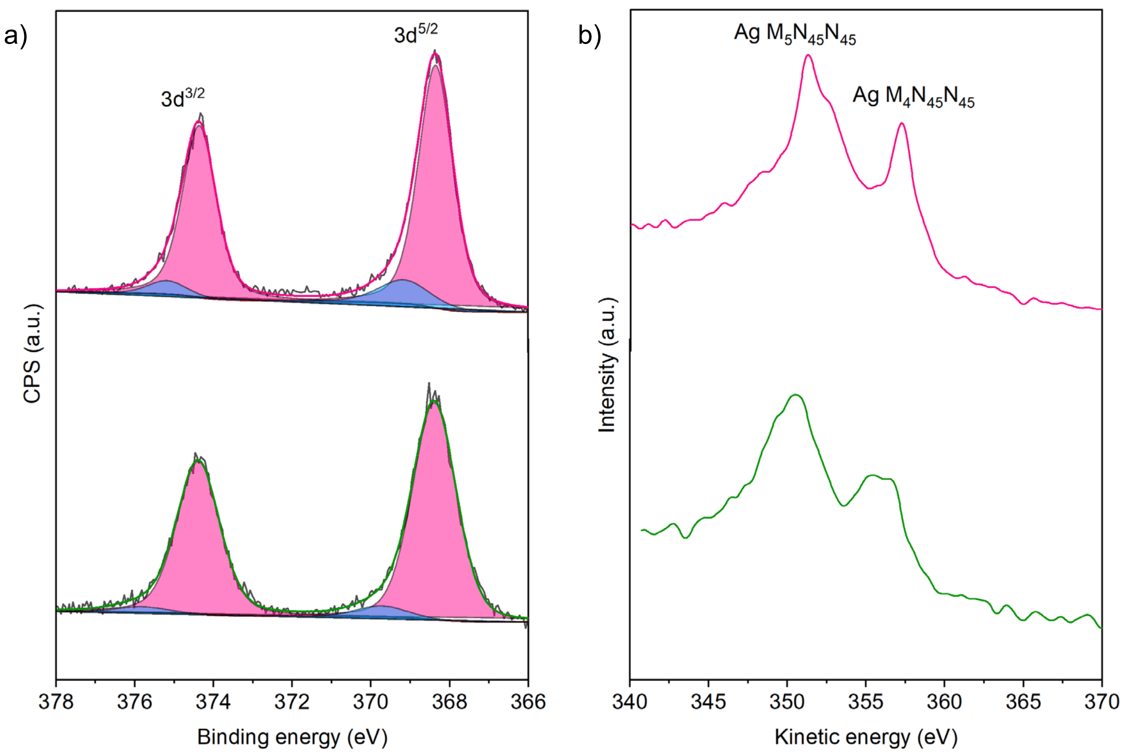

Figure S1: a) High resolution XPS spectra of Ag 3d and b) AES spectra of Ag MNN region for a-C:H:Ag (top) and a-C:H:AgO (bottom).

Water contact angle (WCA) images are shown in **Figure S2**. The addition of the CF_x layer is evidenced by an increase on the WCA regardless of the duty cycle used on a-C:H, a-C:H:Ag, and a-C:H:AgO samples.

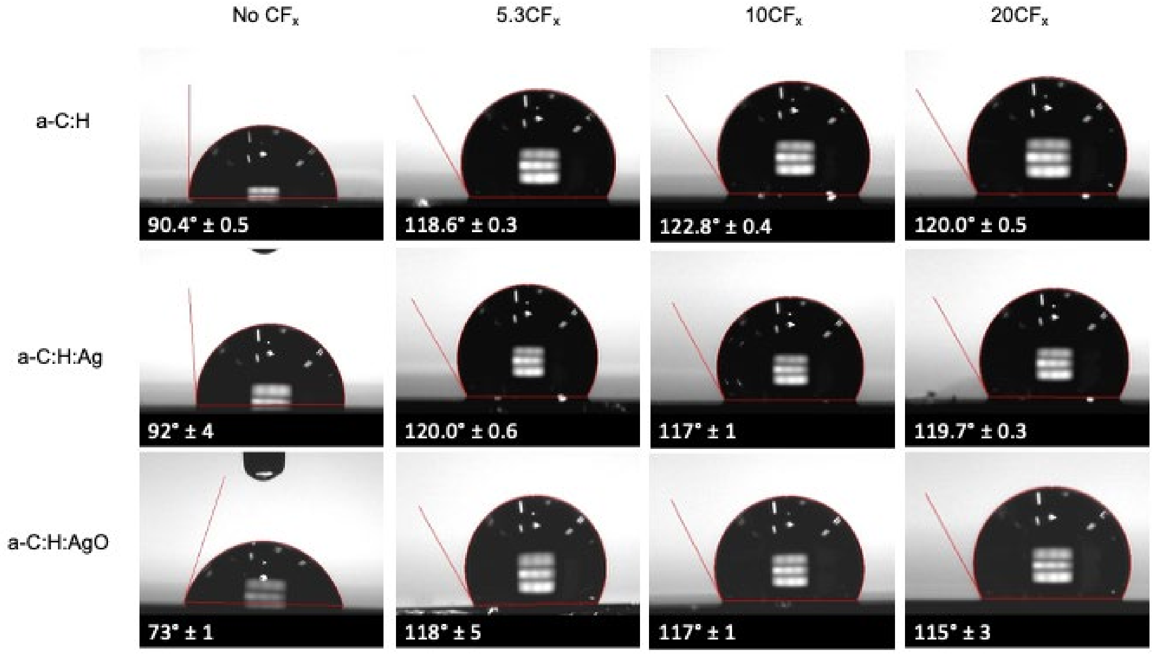

Figure S2: WCA images of the uncoated and CF_x coated a-C:H samples depending on the duty cycle used and their respective WCA values.

AFM was used to study the particle size distribution, surface morphology and roughness (**Figure S3**). Depending on the oxidation state of the target used, Ag and AgO NPs displayed different particle size distributions. Ag NPs exhibits a particle size average of 27 ± 5 nm and a normal distribution, whereas the histogram of AgO NPs yields a particle size average of 23 ± 5 nm and a bimodal size distribution, with two different modes at 20 nm and 33 nm. It is worth mentioning a higher presence of particles with sizes bigger than 30 nm for the a-C:H:AgO surface.

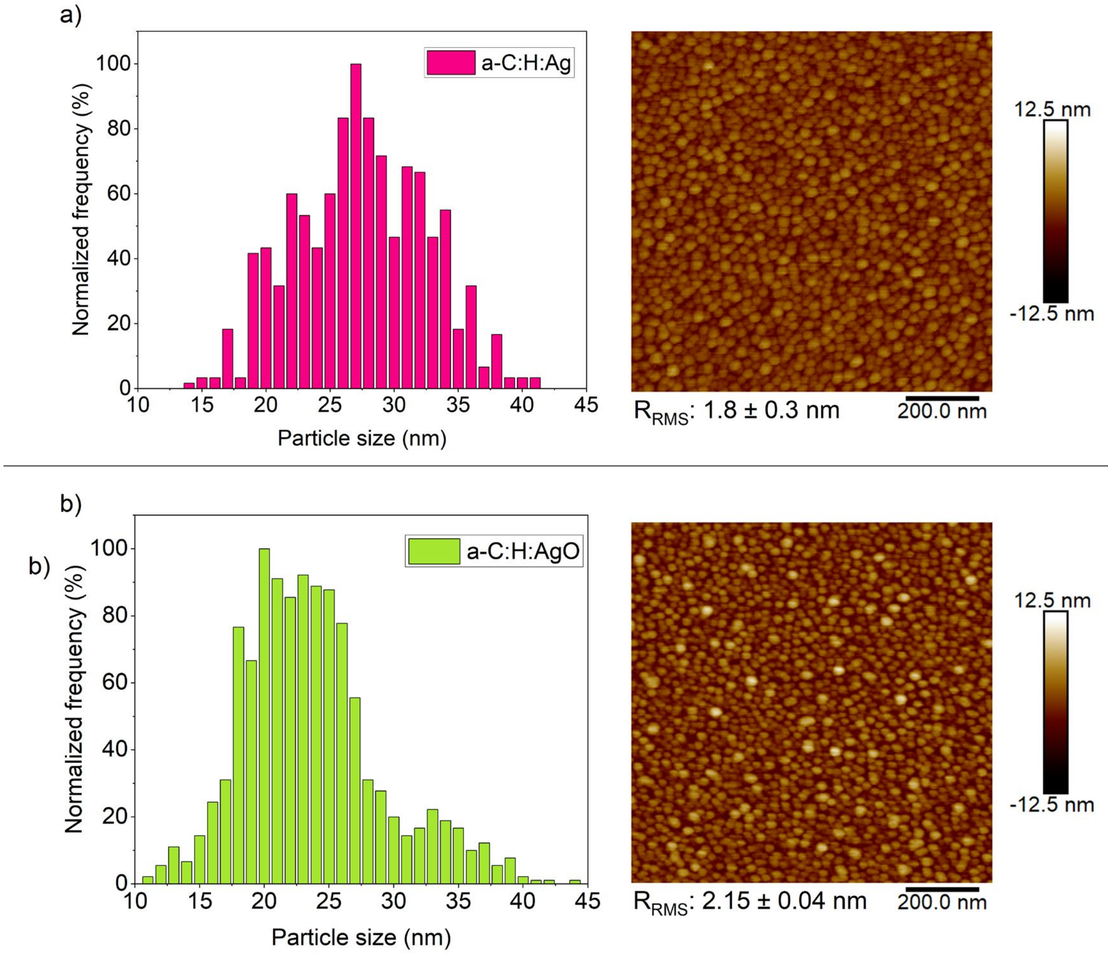

Figure S3: Particle size distribution and AFM images of a) a-C:H:Ag and b) a-C:H:AgO coatings before CF_x deposition.

The antibacterial activity of the 5.3CF$_x$/a-C:H:Ag and 5.3CF$_x$/a-C:H:AgO samples was investigated using *E. coli* after 4 h (**Figure S4**). The results show that 5.3CFx/a-C:H:AgO exhibited significantly higher bacterial inhibition with 63% in comparison with 46% inhibition for 5.3CFx/a-C:H:Ag sample.

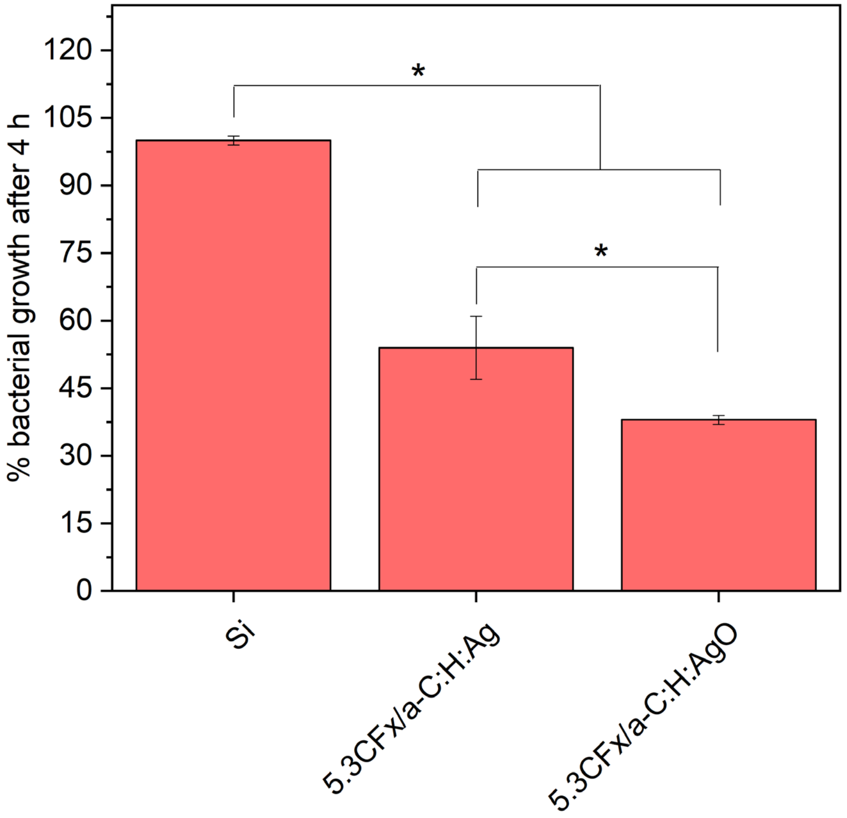

Figure S4: Percentage of *E. coli* growth after 4 h in contact with Si (as negative control), 5.3CF$_x$/a-C:H:Ag and 5.3CF$_x$/a-C:H:AgO ($p < 0.05$).
